# A systematic review and network meta analysis of first-line immunotherapy for advanced urothelial carcinoma

**DOI:** 10.20517/cdr.2026.22

**Published:** 2026-06-05

**Authors:** Yang Liu, Yuxuan Song, Jilin Wu, Jincong Li, Wenbo Yang, Yiqing Du, Caipeng Qin, Tao Xu

**Affiliations:** Department of Urology, Peking University People’s Hospital, Beijing 100044, China.; ^#^Authors contributed equally to this work.

**Keywords:** Urothelial carcinoma, first-line therapy, immune checkpoint inhibitor, antibody–drug conjugate

## Abstract

**Aim:** To compare the efficacy and safety of first-line systemic therapies for advanced urothelial carcinoma (UC) and identify the relative benefit–risk profiles of available regimens.

**Methods:** We conducted a systematic review and Bayesian random-effects network meta-analysis of randomized controlled trials evaluating first-line systemic therapies for advanced UC (INPLASY2025100112). PubMed, Embase, and the Cochrane Library were searched from inception to November 30, 2025. Time-to-event outcomes were summarized as hazard ratios (HRs) with 95% credible intervals (CrIs) for overall survival (OS) and progression-free survival (PFS), and binary outcomes were summarized as odds ratios (ORs) with 95%CrIs for objective response rate (ORR) and grade ≥ 3 adverse events (AEs).

**Results:** Thirteen trials comparing 10 regimens were included. Immune checkpoint inhibitor plus antibody–drug conjugate (ICI+ADC) was associated with the largest estimated improvements in OS, PFS, and ORR (OS: HR 0.47, 95%CrI 0.37-0.63; PFS: HR 0.45, 95%CrI 0.33-0.63; ORR: OR 2.70, 95%CrI 1.60-4.60) and showed consistent improvements across both cisplatin-eligible and cisplatin-ineligible patients. ICI plus chemotherapy (ICI+Chemo) also improved OS (HR 0.83, 95%CrI 0.70-0.96) and PFS (HR 0.78, 95% CrI 0.63-0.95). ICI monotherapy was associated with the lowest odds of grade ≥ 3 AEs (OR 0.11, 95%CrI 0.03-0.44) but ranked lowest for efficacy.

**Conclusion:** ICI+ADC may represent a promising first-line option when available. ICI+Chemo remains an appropriate alternative, whereas ICI monotherapy may be reserved for patients unsuitable for combination therapy.

## INTRODUCTION

Urothelial carcinoma (UC) is the predominant histologic subtype of malignancies arising in the urinary tract, accounting for more than 90% of cases^[1]^. Despite advances in detection and management, outcomes for advanced disease remain poor^[2]^. In the first-line setting for locally advanced or metastatic UC (la/mUC), cisplatin-based combination chemotherapy remains the backbone of systemic therapy^[3,4]^. However, durable disease control remains limited because of intrinsic and acquired treatment resistance^[5,6]^. This resistance often manifests clinically as early progression and limited long-term benefit. Moreover, up to half of patients are ineligible for cisplatin because of renal dysfunction or comorbidities^[4,7]^. These patients often receive less effective carboplatin-based regimens, highlighting the need for more effective first-line strategies.

In recent years, immune checkpoint inhibitors (ICIs) targeting programmed death-1 (PD-1) and its ligand (PD-L1) have reshaped the treatment landscape of UC. Multiple PD-1/PD-L1 inhibitors have received regulatory approval in the second-line setting and as first-line options for cisplatin-ineligible patients, supporting a transition beyond chemotherapy-only strategies^[8-10]^.

With the advent of ICIs, multiple large phase III randomized controlled trials (RCTs), such as IMvigor130^[11]^, KEYNOTE-361^[12]^, DANUBE^[13]^, and CheckMate 901^[14]^, have evaluated the role of ICIs alone or in combination with chemotherapy in the first-line setting. Although these studies did not consistently demonstrate overall survival (OS) superiority in the intention-to-treat population, they provided comparative evidence across major treatment strategies. More recently, the EV-302 trial showed that enfortumab vedotin (EV) plus pembrolizumab significantly improved OS and progression-free survival (PFS) compared with chemotherapy^[15]^. These findings contributed to updated guideline endorsements and reshaped expectations for first-line treatment. Collectively, these trials reflect the increasing evaluation of ICI-based combinations in the first-line setting. However, the durability of benefit remains variable across regimens. In addition, treatment outcomes may differ across biological subgroups. In FGFR-altered UC, selective pan-FGFR inhibitors may yield higher objective response rates (ORRs) than ICI or chemotherapy^[16]^. However, they have not shown a survival benefit and may be associated with more grade ≥ 3 adverse events (AEs). Recent evidence also suggests that FGFR3-mutated tumors display an immunosuppressive microenvironment and diminished response to checkpoint blockade^[17]^. These observations further highlight the biological heterogeneity underlying treatment outcomes. Accordingly, an updated synthesis incorporating recent randomized trials and a broader comparative evidence framework remains clinically relevant.

Despite these advances, direct head-to-head comparisons across the full spectrum of available first-line systemic therapies remain lacking. Traditional pairwise meta-analyses are limited when most regimens have not been compared directly. Previous network meta-analysis (NMA) have evaluated first-line systemic therapy for metastatic UC^[18]^, but important evidence gaps remain. In this context, we conducted a systematic review and NMA of RCTs evaluating first-line systemic therapies for patients with advanced or metastatic UC.

## METHODS

### Search strategy

This systematic review and NMA was conducted in accordance with the preferred reporting items for systematic reviews and meta-analyses (PRISMA) statement. The study protocol was registered in the International Platform of Registered Systematic Review and Meta-analysis Protocols (INPLASY; registration number: INPLASY2025100112).

We performed a comprehensive literature search using PubMed, Embase, and the Cochrane Library. Detailed search strategies for all databases were provided in Supplementary Table 1. Additionally, the reference lists of included articles and relevant reviews were screened to identify any additional eligible studies. Two investigators independently screened titles and abstracts, reviewed full texts for eligibility, and documented exclusion reasons. Disagreements were resolved through discussion with a third author.

### Inclusion and exclusion criteria

We included RCTs investigating first-line systemic treatments for patients with la/mUC. Eligible interventions included chemotherapy, ICIs, their combinations, or novel agents such as antibody–drug conjugates (ADCs). Comparators could be any other active treatment, including chemotherapy, ICIs or placebo. Studies were required to report at least one relevant outcome, including OS, PFS, ORR, or grade ≥ 3 AEs. Trials were excluded if they were single-arm, retrospective, non-randomized, or lacked key outcome data. For studies with overlapping populations, the most complete and up-to-date data were retained for analysis.

### Data extraction

Two reviewers independently extracted and cross-validated study-level data from each eligible trial. Extracted variables included trial name, first author, year of publication, study period, study design, number of centers and countries, sample size per treatment arm, and comparator details. For each included RCT, outcomes of interest were OS, PFS, ORR, and grade ≥ 3 AEs. When available, subgroup data for cisplatin-eligible patients were also extracted to enable stratified survival analysis.

### Quality assessment

The risk of bias (RoB) was assessed using the Cochrane Collaboration’s RoB tool for RCTs^[19]^.

### Statistical analysis

NMA was performed in a Bayesian framework using R software (version 4.5.0) with the gemtc (version 1.1.0) and rjags (version 4.17) packages. Hazard ratios (HRs) and 95% credible intervals (CrIs) were used for time-to-event outcomes (OS and PFS), and odds ratios (ORs) and 95%CrIs were used for binary outcomes (ORR and grade ≥ 3 AEs). When HRs were not directly reported, values were estimated from Kaplan–Meier curves using established methods^[20]^.

For time-to-event outcomes, analyses were based on log(HR) values and their corresponding standard errors. Bayesian random-effects consistency models were fitted using a normal likelihood and identity link. For binary outcomes, Bayesian random-effects consistency models were fitted in gemtc using a binomial likelihood and logit link. Four Markov chains were used, with 5,000 adaptation iterations and 20,000 sampling iterations. Between-study heterogeneity was modeled as a common random-effects standard deviation (tau). The default prior setting implemented in gemtc was used. Network geometry was visualized to assess the structure of treatment comparisons, and treatments were ranked using the surface under the cumulative ranking curve (SUCRA). Node-splitting analyses were not performed in the present study.

### Agreement across endpoints

To assess agreement across endpoints, we applied the pairwise-combination logic of the previously reported rationality-congruence assessment (RCA) framework^[21]^ to four clinical endpoints in the present study. Endpoint directions were coded a priori as favorable (+1), neutral or unreported (0), or unfavorable (-1). Favorable directions were defined as HR < 1 for OS and PFS, OR < 1 for grade ≥ 3 AEs, and OR > 1 for ORR. The primary summary was an agreement score calculated by assigning +1 to each endpoint pair showing concordant benefit (+, +) and -1 to each pair showing concordant harm (-, -) across the following prespecified pairs: (OS, PFS), (OS, ORR), (OS, AEs), (PFS, ORR), (PFS, AEs), and (ORR, AEs) (range, -6 to +6). As a sensitivity analysis, the same rules were applied using two-sided 95%CrIs to define favorable or unfavorable directions. We also report two secondary summaries: (i) a stricter agreement score, which subtracts discordant pairs containing an unfavorable direction (+/- or 0/- pairs) from the primary agreement score (range, -6 to +6); and (ii) a directional net score, defined as the sum of the four endpoint directions for each regimen (range, -4 to +4).

## RESULTS

### Search results

The literature search yielded 1,582 unique records. After removing 13 duplicates, 1,569 records were screened, and 1,546 were excluded due to irrelevance to the study question. Twenty-three full-text articles were assessed for eligibility, and 13 RCTs were included in the final NMA [Supplementary Figure 1]. The detailed characteristics of these included studies are summarized in Table 1. The RoB assessment results for the included RCTs are presented in Supplementary Figure 2. Overall, the included trials were of acceptable methodological quality, with low RoB in most domains. Ten first-line treatment strategies were included in this NMA, encompassing chemotherapy monotherapy (Chemo), chemotherapy doublets (Chemo+Chemo), chemotherapy combined with tyrosine kinase inhibitors (Chemo+TKI), ICI, ICI combined with chemotherapy (ICI+Chemo), dual ICI therapy (ICI+ICI), ICI combined with TKIs (ICI+TKI), ICI combined with ADCs (ICI+ADCs), ICI combined with poly(ADP-ribose) polymerase inhibitors (ICI+PARPi) and ADC. A comparison between the present study and previous published meta-analyses is provided in Supplementary Table 2.

**Table 1:** Detailed baseline characteristics of the included 13 RCTs

**Trial name/ID**	**First author**	**Year**	**Study period**	**Center**	**Phase**	**Median follow up time (months)**	**Outcome**	**No. of patients (treatments)**	**No. of patients** **(control)**
IMvigor130^[11]^	Galsky M. D.	2020	2016-2018	Multicenter	III	11.8	OS, PFS, ORR, AEs	362 (Atezolizumab monotherapy) 400 (Atezolizumab + Chemotherapy)	451 (Chemotherapy)
KEYNOTE-361^[12]^	Powles T.	2021	2016-2018	Multicenter	III	31.7	OS, PFS, ORR, AEs	307 (Pembrolizumab) 351 (Pembrolizumab + Chemotherapy)	352 (Chemotherapy)
DANUBE^[13]^	Powles T.	2020	2015-2017	Multicenter	III	41.2	OS, ORR, AEs	346 (Durvalumab) 342 (Durvalumab + Tremelimumab)	344 (Chemotherapy)
EV-302^[15,22]^	Powles T.	2024	2021-2023	Multicenter	III	17.2	OS, PFS, ORR, AEs	442 (EV + Pembrolizumab)	444 (Chemotherapy)
CheckMate 901^[14]^	van der Heijden M. S.	2023	2018-2022	Multicenter	III	33.6	OS, PFS, ORR, AEs	304 (Nivolumab + Chemotherapy)	304 (Chemotherapy)
BAYOU^[23]^	Rosenberg J. E.	2023	2018-2019	Multicenter	II	9.8	OS, PFS, ORR, AEs	94 (Durvalumab + Olaparib)	94 (Durvalumab + Placebo)
VINGEM^[24]^	Holmsten K.	2020	2014-2018	Multicenter	II	21	OS, PFS, ORR	29 (Vinflunine + Gemcitabine)	30 (Gemcitabine + Cisplatin)
KEYNOTE-672^[25]^	Necchi A.	2024	2017-2018	Multicenter	III	2.1	ORR, AEs	44 (Epacadostat + Pembrolizumab)	49 (Placebo + Pembrolizumab)
ChiCTR1900022615^[26]^	An X.	2024	2019-2021	Single center	III	11.4	OS, PFS, ORR	26 (Gemcitabine + nab-Paclitaxel)	28 (Gemcitabine + Carboplatin)
TOUCAN^[27]^	Jones R.	2020	2011-2014	Multicenter	II	NA	OS, PFS, ORR, AEs	40 (Gemcitabine + Carboplatin + Vandetanib)	42 (Gemcitabine + Carboplatin + Placebo)
LEAP-011^[28]^	Matsubara N.	2024	2019-2021	Multicenter	III	12.8	OS, PFS, ORR, AEs	245 (Lenvatinib + Pembrolizumab)	242 (Placebo + Pembrolizumab)
EV-103 cohort K^[29]^	O’Donnell P. H.	2023	2020-2022	Multicenter	Ib/II	14.8	OS, PFS, ORR, AEs	76 (EV + Pembrolizumab)	73 (EV monotherapy)
COACH/KCSG GU10-16^[30]^	Park I.	2020	2011-2017	Multicenter	II	37.8	OS, PFS, ORR	40 (Gemcitabine + Oxaliplatin)	39 (Gemcitabine + Carboplatin)

RCTs: Randomized controlled trials; OS: overall survival; PFS: progression-free survival; ORR: objective response rate; AEs: adverse events; EV: enfortumab vedotin; NA: not applicable.

### Overall analysis in all patients

#### OS

**Figure 1:**
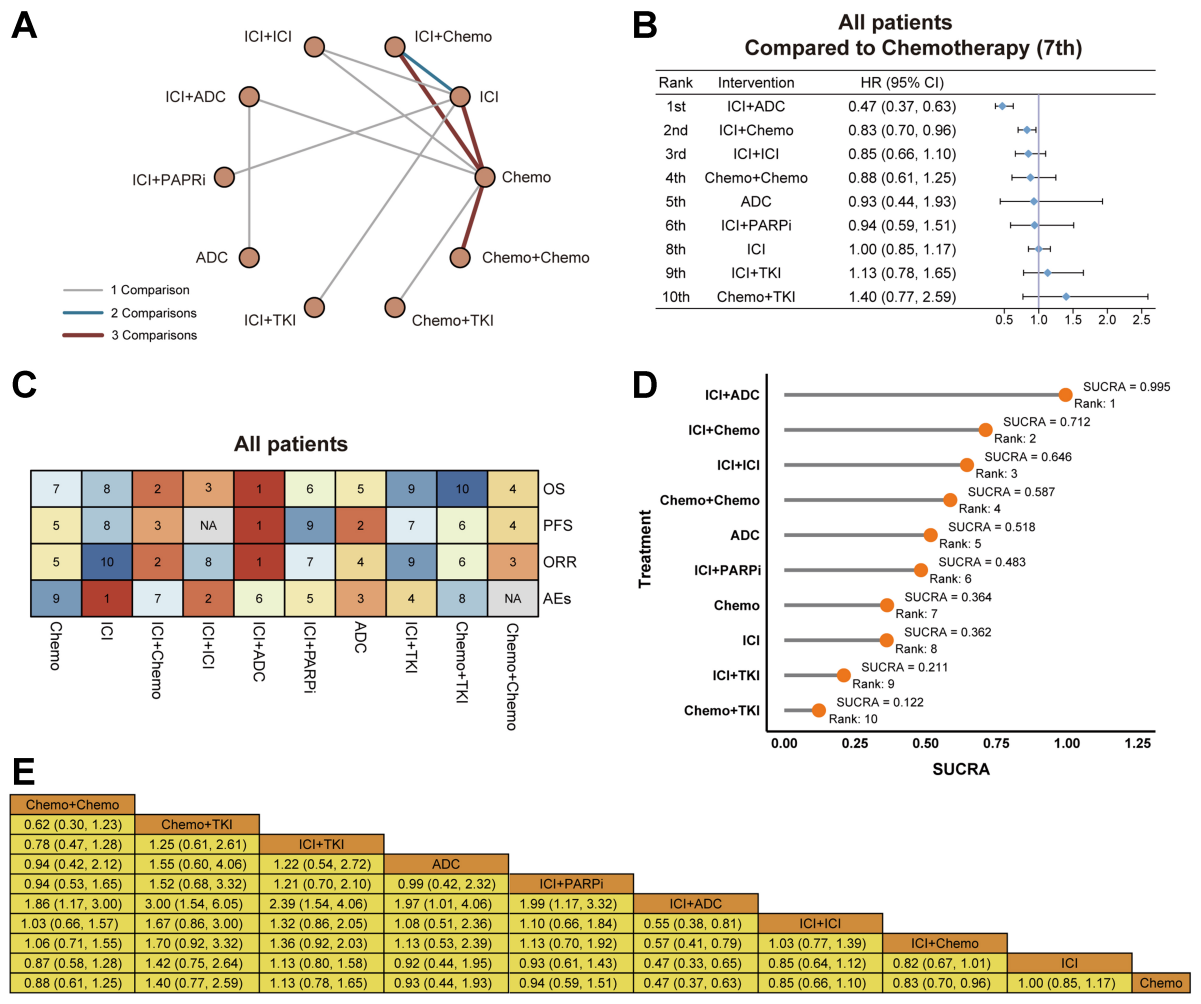
Overall analysis in all patients. (A) Network geometry of OS comparisons among all regimens; edge thickness reflects number of direct comparisons; (B) Forest plot of OS HRs *vs.* chemotherapy (values < 1 favor the experimental regimen); (C) Heatmap summarizing rank positions across OS, PFS, ORR, and AEs (1 = best; higher numbers = worse); (D) SUCRA-based ranking for OS showing the overall hierarchy of regimens; (E) League table of pairwise OS comparisons reporting HRs (95%CrI) between regimens; cells < 1 favor the row treatment over the column treatment. OS: Overall survival; HRs: hazard ratios; PFS: progression-free survival; ORR: objective response rate; AEs: adverse events; SUCRA: surface under the cumulative ranking curve; CrI: credible interval; ICI+ICI: dual immune checkpoint inhibitor therapy; ICI+Chemo: immune checkpoint inhibitor plus chemotherapy; ICI+ADC: immune checkpoint inhibitor plus antibody–drug conjugate; ICI+PARPi: immune checkpoint inhibitor plus poly(ADP-ribose) polymerase inhibitor; Chemo: platinum-based chemotherapy; Chemo+Chemo: chemotherapy doublets; ICI+TKI: immune checkpoint inhibitor plus tyrosine kinase inhibitor; Chemo+TKI: chemotherapy combined with tyrosine kinase inhibitors; CI: confidence interval; NA: not applicable.

OS was evaluated across 12 RCTs^[11-15,23,24,26-30]^ [Figure 1A]. ICI+ADC was associated with improved OS *vs.* chemotherapy (HR 0.47, 95%CrI 0.37-0.63, *P* < 0.0001) [Figure 1B] and ranked first according to SUCRA values [Figure 1C]. ICI+Chemo also demonstrated statistically significant OS improvement over chemotherapy (HR 0.83, 95%CrI 0.70-0.96, *P* = 0.021) [Figure 1B], ranking second [Figure 1C]. Other regimens did not yield statistically significant OS benefits [Figure 1B].

In the SUCRA ranking plot, ICI+ADC ranked first, followed by ICI+Chemo, whereas ICI+TKI and Chemo+TKI ranked lowest [Figure 1D]. The league table showed that only ICI+ADC and ICI+Chemo had 95%CrIs excluding 1 when compared with chemotherapy, whereas most other comparisons had 95%CrIs crossing 1 [Figure 1E].

#### PFS

PFS was evaluated across 11 RCTs^[11,12,14,15,23,24,26-30]^ [Supplementary Figure 3A]. ICI+ADC was associated with improved PFS *vs.* chemotherapy (HR 0.45, 95%CrI 0.33-0.63, *P* < 0.0001) [Supplementary Figure 3B], ranking first among all regimens [Figure 1C]. ICI+Chemo also showed a statistically significant improvement in PFS (HR 0.78, 95%CrI 0.63-0.95, *P* = 0.018) [Supplementary Figure 3B], ranking third [Figure 1C]. Other regimens showed no statistically significant advantage over chemotherapy [Supplementary Figure 3B].

#### ORR

ORR was evaluated across all 13 RCTs^[11-15,23-30]^ [Supplementary Figure 3C]. Only two regimens showed significantly higher response compared to chemotherapy. ICI+ADC ranked first with an OR of 2.70 (95%CrI 1.60-4.60, *P* = 0.0003) [Supplementary Figure 3D], followed by ICI+Chemo (OR 1.40, 95%CrI 1.00-1.90, *P* = 0.04) [Supplementary Figure 3D], which ranked second [Figure 1C]. Other regimens showed no statistically significant difference in ORR compared to chemotherapy [Supplementary Figure 3D].

#### Grade ≥ 3 AEs

Grade ≥ 3 AEs were evaluated across 10 RCTs^[11-15,23,25,27-29]^ [Supplementary Figure 3E]. Using chemotherapy as the reference, Chemo+TKI (OR 1.30, 95%CrI 0.08-20.00, *P* = 0.8522) and ICI+Chemo (OR 0.72, 95%CrI 0.17-3.00, *P* = 0.6537) ranked highest in the SUCRA analysis for grade ≥ 3 AEs [Supplementary Figure 3F]. ICI monotherapy was associated with the lowest odds of grade ≥ 3 AEs (OR 0.11, 95%CrI 0.03-0.44, *P* = 0.0013) and ranked first in the SUCRA analysis for grade ≥ 3 AEs [Supplementary Figure 3F].

#### Agreement across endpoints

In the primary analysis based on point estimates, ICI+ADC, ICI+Chemo, and ADC monotherapy each achieved the maximum agreement score of 6, with favorable directions across OS, PFS, ORR, and grade ≥ 3 AEs [Table 2]. Chemo+Chemo achieved a score of 3, and ICI+ICI achieved a score of 1. ICI monotherapy, ICI+PARPi, and TKI-containing combinations did not achieve positive scores (scores ≤ 0), with inconsistent directions across efficacy and safety endpoints. In the sensitivity analysis based on 95%CrIs, ICI+ADC and ICI+Chemo remained the highest-ranked regimens, each with an agreement score of 3, whereas all other regimens did not achieve positive scores (scores ≤ 0) [Table 3].

**Table 2:** Endpoint directions and agreement summaries by regimen using the point-estimate specification

**Regimen**	***OS_dir_*(PE)**	***PFS_dir_*(PE)**	***ORR_dir_*(PE)**	***AEs_dir_*(PE)**	***RCA_lite_*(PE)**	***RCA_cob_*(PE)**	***RCA_scob_*(PE)**
ICI+Chemo	1	1	1	1	4	6	6
ICI+ADC	1	1	1	1	4	6	6
ADC monotherapy	1	1	1	1	4	6	6
Chemo+Chemo	1	1	1	NA	3	3	3
ICI+ICI (dual ICI)	1	NA	-1	1	1	1	-1
ICI+PARPi	1	-1	-1	1	0	0	-4
Chemo (reference)	0	0	0	0	0	0	0
ICI monotherapy	0	-1	-1	1	-1	-1	-3
ICI+TKI	-1	-1	-1	1	-2	-3	-6
Chemo+TKI	-1	-1	-1	-1	-4	-6	-6

Favorable directions were defined as HR < 1 for OS and PFS, OR < 1 for AEs, and OR > 1 for ORR. The net score represents the sum of the four endpoint directions (range, -4 to +4). The agreement score was calculated as +1 for each endpoint pair with concordant benefit and -1 for each endpoint pair with concordant harm (range, -6 to +6). The strict agreement score was calculated as +1 for each endpoint pair with concordant benefit and -1 for each discordant pair containing an unfavorable direction (range, -6 to +6). Coding: +1 = favorable, 0 = neutral or unreported, -1 = unfavorable. OS: Overall survival; PFS: progression-free survival; ORR: objective response rate; AEs: adverse events. *_dir_*(PE): endpoint direction under the point estimate rule; RCA: rationality-congruence assessment; ICI+Chemo: immune checkpoint inhibitor plus chemotherapy; ICI+ADC: immune checkpoint inhibitor plus antibody–drug conjugate; Chemo+Chemo: chemotherapy doublets; NA: not applicable; ICI+ICI: dual immune checkpoint inhibitor therapy; ICI+PARPi: immune checkpoint inhibitor plus poly(ADP-ribose) polymerase inhibitor; Chemo: platinum-based chemotherapy; ICI+TKI: immune checkpoint inhibitor plus tyrosine kinase inhibitor; Chemo+TKI: chemotherapy combined with tyrosine kinase inhibitors; HR: hazard ratio; OR: odds ratio.

**Table 3:** Endpoint directions and agreement summaries by regimen using the confidence-interval-based significance specification

**Regimen**	***OS_dir_*(sig)**	***PFS_dir_*(sig)**	***ORR_dir_*(sig)**	***AEs_dir_*(sig)**	***RCA_lite_*(sig)**	***RCA_cob_*(sig)**	***RCA_scob_*(sig)**
ICI+Chemo	1	1	1	0	3	3	3
ICI+ADC	1	1	1	0	3	3	3
ADC monotherapy	0	0	0	0	0	0	0
Chemo+Chemo	0	0	0	NA	0	0	0
ICI+ICI (dual ICI)	0	NA	0	0	0	0	0
ICI+PARPi	0	0	0	0	0	0	0
Chemo (reference)	0	0	0	0	0	0	0
ICI monotherapy	0	0	-1	1	0	0	-3
ICI+TKI	0	0	0	0	0	0	0
Chemo+TKI	0	0	0	0	0	0	0

Favorable directions were defined as upper CrIs < 1 for OS, PFS, and AEs, and lower CrIs > 1 for ORR. The net score represents the sum of the four endpoint directions (range, -4 to +4). The agreement score was calculated as +1 for each endpoint pair with concordant benefit and -1 for each endpoint pair with concordant harm (range, -6 to +6). The strict agreement score was calculated as +1 for each endpoint pair with concordant benefit and -1 for each discordant pair containing an unfavorable direction (range, -6 to +6). Coding: +1 = favorable, 0 = neutral or unreported, -1 = unfavorable. OS: Overall survival; PFS: progression-free survival; ORR: objective response rate; AEs: adverse events; *_dir_*(sig): endpoint direction under the significance rule based on two-sided 95%CrIs; RCA: rationality-congruence assessment; ICI+Chemo: immune checkpoint inhibitor plus chemotherapy; ICI+ADC: immune checkpoint inhibitor plus antibody–drug conjugate; Chemo+Chemo: chemotherapy doublets; NA: not applicable; ICI+ICI: dual immune checkpoint inhibitor therapy; ICI+PARPi: immune checkpoint inhibitor plus poly(ADP-ribose) polymerase inhibitor; Chemo: platinum-based chemotherapy; ICI+TKI: immune checkpoint inhibitor plus tyrosine kinase inhibitor; Chemo+TKI: chemotherapy combined with tyrosine kinase inhibitors; CrIs: credible intervals.

### Cisplatin-eligible patients

**Figure 2:**
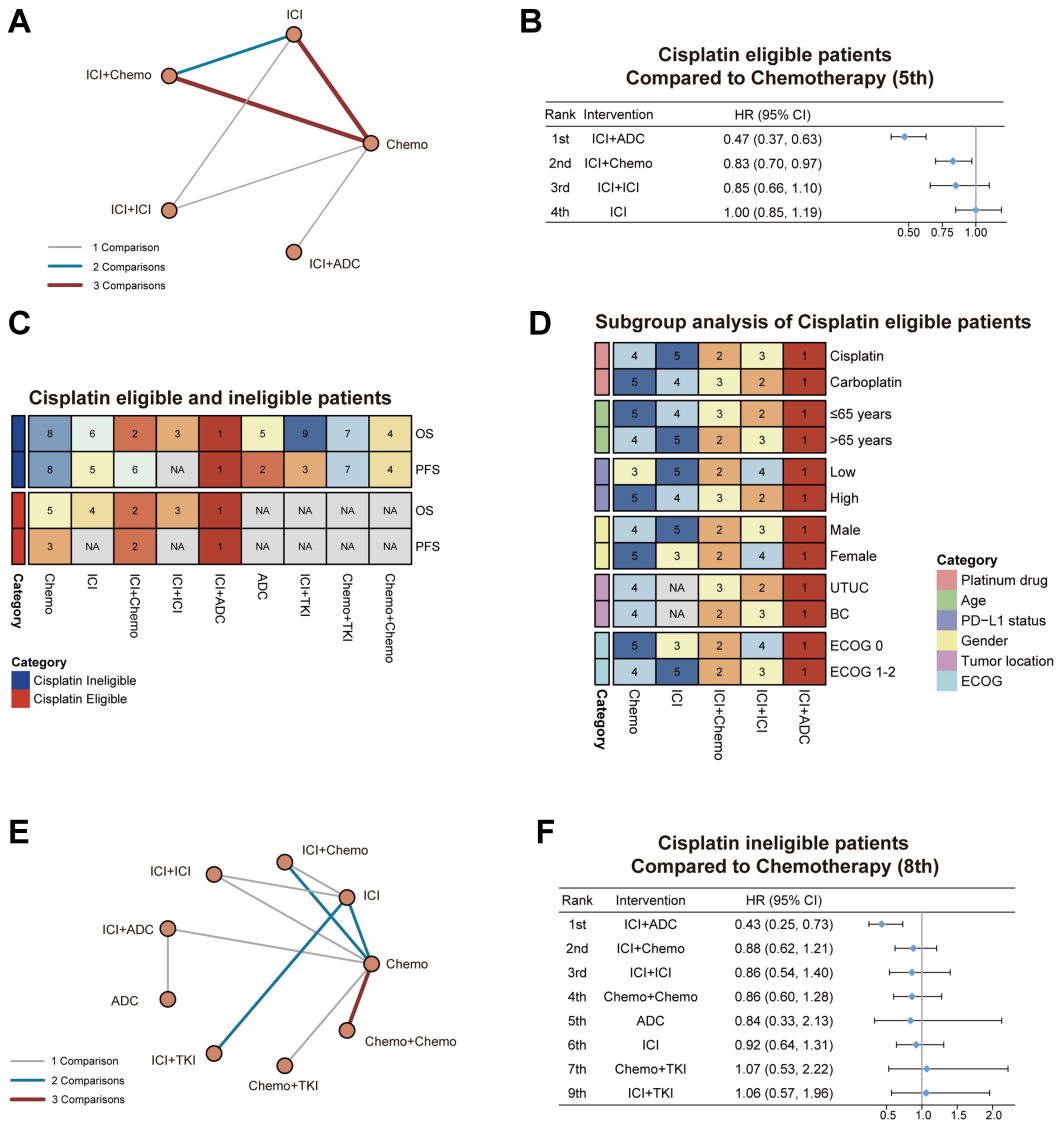
OS by cisplatin eligibility with subgroup ranking. (A) Network geometry for cisplatin-eligible patients; (B) Forest plot of OS HRs *vs.* chemotherapy in cisplatin-eligible patients; (C) Heatmap of rank positions for OS and PFS in cisplatin-eligible and cisplatin-ineligible patients; (D) Heatmap of subgroup OS rankings within the cisplatin-eligible population (platinum drug, age, PD-L1 status, tumor location, gender, ECOG); (E) Network geometry for cisplatin-ineligible patients; (F) Forest plot of OS HRs *vs.* chemotherapy in cisplatin-ineligible patients. OS: Overall survival; HRs: hazard ratios; PFS: progression-free survival; PD-L1: programmed death-ligand 1; ECOG: Eastern Cooperative Oncology Group; ICI: immune checkpoint inhibitor; ICI+Chemo: immune checkpoint inhibitor plus chemotherapy; Chemo: platinum-based chemotherapy; ICI+ICI: dual immune checkpoint inhibitor therapy; ICI+ADC: immune checkpoint inhibitor plus antibody–drug conjugate; CI: confidence interval; NA: not applicable; ICI+TKI: immune checkpoint inhibitor plus tyrosine kinase inhibitor; Chemo+TKI: chemotherapy combined with tyrosine kinase inhibitors; Chemo+Chemo: chemotherapy doublets; UTUC: upper tract urothelial carcinoma; BC: bladder cancer.

A total of five RCTs reported outcomes in cisplatin-eligible patients^[11-15]^ [Figure 2A]. ICI+ADC was associated with improved OS *vs.* chemotherapy (HR 0.47, 95%CrI 0.37-0.63, *P* < 0.0001) [Figure 2B] and ranked first [Figure 2C]. ICI+Chemo also demonstrated a statistically significant OS improvement (HR 0.83, 95%CrI 0.70-0.97, *P* = 0.025) [Figure 2B], ranking second [Figure 2C]. In contrast, both ICI+ICI (HR 0.85, 95%CrI 0.66-1.10, *P* = 0.21) and ICI monotherapy (HR 1.00, 95%CrI 0.85-1.19, *P* = 1.00) [Figure 2B] showed CrIs crossing 1, indicating no statistically conclusive OS benefit in this subgroup. PFS was reported in three RCTs [Supplementary Figure 4A]. ICI+ADC ranked first (HR 0.45, 95%CrI 0.27-0.75, *P* = 0.0021), followed by ICI+Chemo (HR 0.78, 95%CrI 0.58-1.04, *P* = 0.095) [Supplementary Figure 4B].

#### Exploratory subgroup analyses

Among patients eligible for cisplatin-based chemotherapy, subgroup analyses across 12 clinical strata showed that ICI+ADC ranked first in all subgroups [Figure 2D]. ICI+Chemo ranked second in 8 of the 12 subgroups, including patients younger than 65 years, those with high PD-L1 expression, and those with an Eastern Cooperative Oncology Group (ECOG) performance status of 1 or greater. ICI+ICI ranked 3rd or 4th across most subgroups, with the lowest HR observed in the age < 65 subgroup. By contrast, its ranking was lower among patients with low PD-L1 expression or those with bladder cancer (BC) subtypes. ICI monotherapy ranked lowest in 9 subgroups, with HRs ≥ 1.0 in populations defined by cisplatin eligibility, low PD-L1 expression, female sex, and BC subtype. Chemotherapy was used as the reference and ranked last only in the ECOG = 0 subgroup. For PFS, a similar pattern was observed. ICI+ADC ranked first across all 12 subgroups, followed by ICI+Chemo and ICI+ICI [Supplementary Figure 4C]. ICI monotherapy consistently ranked lowest, without a higher ranking in any subgroup [Supplementary Figure 4C].

#### Treatment comparisons

**Figure 3:**
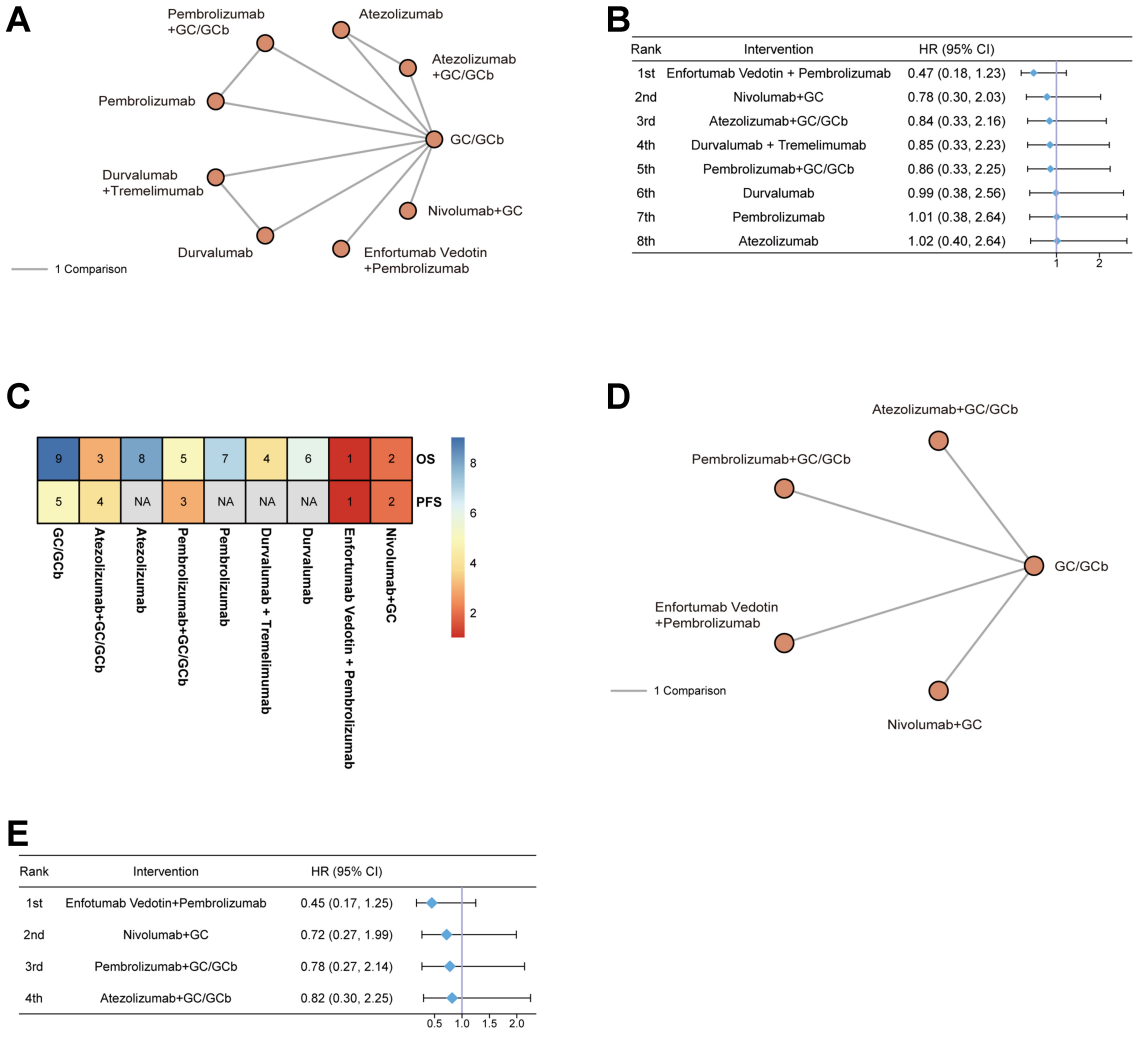
Regimen comparisons within cisplatin-eligible patients. (A) Network geometry for OS; (B) Forest plot of OS HRs *vs.* chemotherapy with the corresponding ranks; (C) Heatmap of rank positions for OS and PFS across regimens in the cisplatin-eligible subgroup; (D) Network geometry for PFS; (E) Forest plot of PFS HRs *vs.* chemotherapy. OS: Overall survival; HRs: hazard ratios; PFS: progression-free survival; GC/GCb: gemcitabine plus cisplatin/gemcitabine plus carboplatin; CI: confidence interval; NA: not applicable.

We further performed an NMA of the five RCTs reporting cisplatin-eligible patients to compare specific treatment regimens [Figure 3]. For OS, the network geometry indicated that gemcitabine plus cisplatin/gemcitabine plus carboplatin (GC/GCb) served as the primary comparator across most included studies [Figure 3A]. Although no statistically significant differences were detected among the regimens [Figure 3B], SUCRA-based ranking probabilities were also summarized. EV plus pembrolizumab ranked highest, followed by nivolumab in combination with GC/GCb and atezolizumab in combination with GC/GCb, whereas durvalumab-containing regimens and pembrolizumab monotherapy showed intermediate ranking probabilities. By contrast, atezolizumab monotherapy and standard chemotherapy (GC/GCb) consistently ranked lowest [Figure 3C].

For PFS, the network geometry similarly showed GC/GCb as the central comparator [Figure 3D]. No statistically significant differences were observed across regimens [Figure 3E], but the ranking probabilities were consistent with OS, with EV plus pembrolizumab occupying the top position and GC/GCb chemotherapy ranked lowest.

### Cisplatin-ineligible patients

In total, 11 studies reported OS in cisplatin-ineligible patients [Figure 2E]. Among them, 4 RCTs provided subgroup data extracted from broader populations (at least carboplatin-eligible)^[11,12,13,15]^, resulting in overlap with this group, while 7 trials specifically enrolled cisplatin-ineligible patients as their study population^[24-30]^. One additional study was excluded because of inconsistent definitions regarding cisplatin eligibility^[23]^. Of these, 10 RCTs reported PFS outcomes, while KEYNOTE-672^[25]^ did not provide PFS results [Supplementary Figure 4D].

In cisplatin-ineligible patients, ICI+ADC consistently ranked first in both OS and PFS [Figure 2C]. ICI+ADC showed a significant OS benefit with a HR of 0.43 (95%CrI 0.25-0.73, *P* = 0.0020) [Figure 2F] and a PFS benefit with HR 0.43 (95%CrI 0.27-0.65, *P* = 0.0001) [Supplementary Figure 4E]. For OS, ICI+Chemo (HR 0.88, 95%CrI 0.62-1.21, *P* = 0.45) and ICI+ICI (HR 0.86, 95%CrI 0.54-1.40, *P* = 0.54) ranked second and third, respectively. Other regimens did not show statistically conclusive benefit [Figure 2F]. Similarly, in PFS analysis, ADC monotherapy ranked second (HR 0.59, 95%CrI 0.30-1.17, *P* = 0.13) [Supplementary Figure 4E]. Other regimens did not show statistically conclusive benefit [Supplementary Figure 4E].

## DISCUSSION

This NMA synthesized evidence from 13 RCTs evaluating 10 first-line regimens for advanced or metastatic UC. Across the overall population, ICI+ADC demonstrated the most consistent and substantial benefit in OS, PFS, and ORR, ranking first in nearly all analyses. ICI+Chemo also showed significant but more modest improvements, while ICI+ICI provided variable benefit without statistical superiority. In contrast, ICI monotherapy and TKI-based combinations failed to outperform chemotherapy. Safety analysis indicated that ICI monotherapy had the most favorable toxicity profile, though at the cost of limited efficacy, whereas combination regimens, especially ICI+ADC and ICI+Chemo, were associated with higher rates of grade ≥ 3 AEs. Compared with previous published meta-analyses, the present study incorporates a more contemporary first-line evidence base, including ADC-era randomized trials such as EV-302 and other recently reported studies. We also evaluated outcomes separately in cisplatin-eligible and cisplatin-ineligible patients and included an exploratory cross-endpoint consistency analysis to jointly interpret efficacy and safety signals. These additions may provide more clinically relevant information for treatment selection in current practice.

The advantage of ICI+ADC is consistent with the EV-302 trial, which established EV plus pembrolizumab as a key first-line option. Our results extend this evidence by showing consistent benefit in both cisplatin eligible and cisplatin ineligible subgroups. Similar patterns were observed across age, ECOG performance status, and PD-L1 strata. The signal in cisplatin ineligible and PD-L1 low patients is clinically relevant because durable benefit is often limited in these settings. The ICI+ADC strategy is also supported by the RC48-C016 trial^[31]^. This study enrolled HER2 positive patients and evaluated disitamab vedotin plus toripalimab. The observed survival benefit provides additional support for ICI+ADC combinations across different ADC platforms.

ICI+Chemo showed meaningful benefit in several subgroups, including PD-L1 high and ECOG 1 or higher patients, which is consistent with signals reported in IMvigor130. In contrast, ICI+ICI did not provide clear benefit in the overall population, which is in line with the negative DANUBE results. ICI monotherapy ranked low across most endpoints, supporting its limited role as first-line treatment in unselected patients. These patterns also fit a resistance framework. Single agent ICI may fail to control early treatment failure in tumors with primary immune resistance. Combination strategies may reduce early nonresponse by adding a nonoverlapping antitumor component, but they increase toxicity.

These findings inform first-line treatment selection in advanced UC. ICI+ADC showed the strongest efficacy profile and can be considered the preferred option when available. However, the real-world adoption of ICI+ADC combinations may be constrained by higher treatment costs, reimbursement barriers, and limited accessibility in some healthcare settings. Therefore, although these regimens showed the most favorable efficacy profile, financial toxicity and availability remain important considerations in routine clinical decision-making. This is relevant to the scope of cancer drug resistance because early progression and short-lived responses represent resistance related treatment failure in routine care. Regimens that improve both PFS and ORR are more likely to delay early treatment failure at the population level. ICI+Chemo remains an appropriate alternative when ADCs are unavailable or unsuitable. This may be most relevant in groups with higher risk of early failure, such as patients with ECOG 1 or higher or PD-L1 high tumors. ICI+ICI may be considered for selected patients, but current evidence does not support broad use. ICI monotherapy showed the most favorable safety profile, but it ranked low for efficacy outcomes. Its role may be limited to patients who cannot tolerate combination therapy or who prioritize safety. Treatment decisions should balance efficacy and toxicity. They should also consider the goal of delaying resistance related treatment failure.

We further evaluated agreement across OS, PFS, ORR, and grade ≥ 3 AEs using the previously described framework. Agreement across endpoints may support a more consistent overall benefit–risk pattern, whereas inconsistency across endpoints may indicate efficacy–safety trade-offs. In our NMA, ICI+ADC and ICI+Chemo showed consistently favorable directions across endpoint pairs, in line with their leading SUCRA ranks. Chemo+Chemo showed partial agreement across endpoints, whereas ICI monotherapy and TKI-containing combinations showed greater inconsistency across endpoints. When we required CrIs to exclude the null, agreement scores decreased, but the overall hierarchy remained similar, with ICI+ADC and ICI+Chemo remaining top ranked. This framework complements SUCRA and league tables by summarizing whether efficacy and safety endpoints move in the same direction, but it does not account for effect size, baseline risk, or patient utility. Future work should incorporate patient-reported outcomes (PROs) and individual participant data (IPD) with explicit utility weighting to better reflect clinical value and resistance-related treatment failure.

Several limitations merit consideration. First, we included only RCTs, but heterogeneity in trial design, eligibility criteria, and patient characteristics may introduce bias and weaken the transitivity assumption of the NMA. In addition, several included studies were phase II trials with relatively small sample sizes. Although these trials were randomized and helped improve network coverage across currently available first-line strategies, their inclusion may have increased uncertainty in some comparisons and treatment rankings. Differences in PD-L1 assays, cutoffs, and endpoint definitions or assessment methods across trials may also affect comparability and represent an additional source of clinical heterogeneity. Second, most regimens were not compared directly, and the HRs for EV-103 cohort K were estimated from the published Kaplan–Meier curves rather than directly reported, which may reduce precision. In addition, KEYNOTE-672 had a short median follow-up of 64 days because enrollment was discontinued early. However, this trial contributed only ORR and grade ≥ 3 AE data and was not included in the OS or PFS analyses. Third, subgroup analyses were constrained by available reporting and should be interpreted cautiously.

Future head-to-head trials are needed to compare leading regimens, particularly ICI+ADC *vs.* ICI+Chemo. Precision oncology approaches that incorporate PD-L1, tumor mutational burden (TMB), molecular subtypes, and immune gene signatures may improve patient selection. Real-world evidence and IPD are likely to be important to validate these findings and inform sequencing. Further work should also examine mechanisms of resistance to ICI, ADC, and combination regimens, as well as optimal treatment duration. Emerging modalities such as bispecific antibodies and cellular therapies may further expand options.

In conclusion, this NMA suggests that ICI+ADC was associated with the most consistent improvement in OS, PFS, and ORR in advanced UC, including cisplatin-eligible and cisplatin-ineligible patients. ICI+Chemo also improves outcomes and remains a practical option when ADCs are unavailable. ICI monotherapy shows limited efficacy despite favorable safety and may be reserved for patients unfit for combination regimens. Further head-to-head trials and biomarker-based approaches are needed to refine first-line selection.

## DECLARATIONS

### Availability of data and materials

The datasets used and analyzed during the current study are available from the corresponding author on reasonable request.

### Copyright

© The Author(s) 2026.

## References

[1] Miyazaki J, Nishiyama H (2017). Epidemiology of urothelial carcinoma. Int J Urol.

[2] Siegel RL, Giaquinto AN, Jemal A (2024). Cancer statistics, 2024. CA Cancer J Clin.

[3] von der Maase H, Hansen SW, Roberts JT (2000). Gemcitabine and cisplatin versus methotrexate, vinblastine, doxorubicin, and cisplatin in advanced or metastatic bladder cancer: results of a large, randomized, multinational, multicenter, phase III study. J Clin Oncol.

[4] Galsky MD, Hahn NM, Rosenberg J (2011). A consensus definition of patients with metastatic urothelial carcinoma who are unfit for cisplatin-based chemotherapy. Lancet Oncol.

[5] Rajendran G, Taylor JA 3rd, Woolbright BL (2021). Natural products as a means of overcoming cisplatin chemoresistance in bladder cancer. Cancer Drug Resist.

[6] (2022). Ruiz de Porras V. Natural bioactive compounds: a potential therapeutic strategy to sensitize bladder cancer to cisplatin treatment?. Cancer Drug Resist.

[7] De Santis M, Bellmunt J, Mead G (2012). Randomized phase II/III trial assessing gemcitabine/carboplatin and methotrexate/carboplatin/vinblastine in patients with advanced urothelial cancer who are unfit for cisplatin-based chemotherapy: EORTC study 30986. J Clin Oncol.

[8] (2017). Bellmunt J, de Wit R, Vaughn DJ, et al.; KEYNOTE-045 Investigators. Pembrolizumab as second-line therapy for advanced urothelial carcinoma. N Engl J Med.

[9] (2017). Balar AV, Galsky MD, Rosenberg JE, et al.; IMvigor210 Study Group. Atezolizumab as first-line treatment in cisplatin-ineligible patients with locally advanced and metastatic urothelial carcinoma: a single-arm, multicentre, phase 2 trial. Lancet.

[10] Powles T, Park SH, Caserta C (2023). Avelumab first-line maintenance for advanced urothelial carcinoma: results from the JAVELIN bladder 100 trial after ≥ 2 years of follow-up. J Clin Oncol.

[11] (2020). Galsky MD, Arija JÁA, Bamias A, et al.; IMvigor130 Study Group. Atezolizumab with or without chemotherapy in metastatic urothelial cancer (IMvigor130): a multicentre, randomised, placebo-controlled phase 3 trial. Lancet.

[12] (2021). Powles T, Csőszi T, Özgüroğlu M, et al.; KEYNOTE-361 Investigators. Pembrolizumab alone or combined with chemotherapy versus chemotherapy as first-line therapy for advanced urothelial carcinoma (KEYNOTE-361): a randomised, open-label, phase 3 trial. Lancet Oncol.

[13] (2020). Powles T, van der Heijden MS, Castellano D, et al.; DANUBE study investigators. Durvalumab alone and durvalumab plus tremelimumab versus chemotherapy in previously untreated patients with unresectable, locally advanced or metastatic urothelial carcinoma (DANUBE): a randomised, open-label, multicentre, phase 3 trial. Lancet Oncol.

[14] (2023). van der Heijden MS, Sonpavde G, Powles T, et al.; CheckMate 901 Trial Investigators. Nivolumab plus gemcitabine-cisplatin in advanced urothelial carcinoma. N Engl J Med.

[15] Gupta S, Loriot Y, Van der Heijden MS (2025). Enfortumab vedotin plus pembrolizumab versus chemotherapy in patients with previously untreated locally advanced or metastatic urothelial cancer (EV-302): patient-reported outcomes from an open-label, randomised, controlled, phase 3 study. Lancet Oncol.

[16] Song Y, Du Y, Jiang S, Peng Y, Luo X, Xu T (2025). Efficacy and safety of selective pan-fibroblast growth factor receptor (FGFR) tyrosine kinase inhibitors in FGFR-altered urothelial carcinoma. Pharmacol Res.

[17] Song Y, Peng Y, Qin C (2023). Fibroblast growth factor receptor 3 mutation attenuates response to immune checkpoint blockade in metastatic urothelial carcinoma by driving immunosuppressive microenvironment. J Immunother Cancer.

[18] Chen HL, Chan VW, Tu YK (2021). Immune checkpoints inhibitors and chemotherapy as first-line treatment for metastatic urothelial carcinoma: a network meta-analysis of randomized phase III clinical trials. Cancers.

[19] (2011). Higgins JP, Altman DG, Gøtzsche PC, et al.; Cochrane Bias Methods Group, Cochrane Statistical Methods Group. The Cochrane Collaboration’s tool for assessing risk of bias in randomised trials. BMJ.

[20] Tierney JF, Stewart LA, Ghersi D, Burdett S, Sydes MR (2007). Practical methods for incorporating summary time-to-event data into meta-analysis. Trials.

[21] Mou W, Deng Z, Zhu L (2025). Intratumoral mycobiome heterogeneity influences the tumor microenvironment and immunotherapy outcomes in renal cell carcinoma. Sci Adv.

[22] (2024). Powles T, Valderrama BP, Gupta S, et al.; EV-302 Trial Investigators. Enfortumab Vedotin and Pembrolizumab in Untreated Advanced Urothelial Cancer. N Engl J Med.

[23] Rosenberg JE, Park SH, Kozlov V (2023). Durvalumab plus olaparib in previously untreated, platinum-ineligible patients with metastatic urothelial carcinoma: a multicenter, randomized, phase II trial (BAYOU). J Clin Oncol.

[24] Holmsten K, Jensen NV, Mouritsen LS (2020). Vinflunine/gemcitabine versus carboplatin/gemcitabine as first-line treatment in cisplatin-ineligible patients with advanced urothelial carcinoma: a randomised phase II trial (VINGEM). Eur J Cancer.

[25] Necchi A, Van der Heijden MS, Trukhin D (2024). Pembrolizumab plus either epacadostat or placebo for cisplatin-ineligible urothelial carcinoma: results from the ECHO-307/KEYNOTE-672 study. BMC Cancer.

[26] An X, Xue C, Chen M (2024). Gemcitabine/nab-paclitaxel vs gemcitabine/carboplatin for advanced urothelial carcinoma. BJU Int.

[27] Jones R, Crabb S, Chester J (2020). A randomised Phase II trial of carboplatin and gemcitabine ± vandetanib in first-line treatment of patients with advanced urothelial cell cancer not suitable to receive cisplatin. BJU Int.

[28] Matsubara N, de Wit R, Balar AV (2024). Pembrolizumab with or without lenvatinib as first-line therapy for patients with advanced urothelial carcinoma (LEAP-011): a phase 3, randomized, double-blind trial. Eur Urol.

[29] O’Donnell PH, Milowsky MI, Petrylak DP (2023). Enfortumab vedotin with or without pembrolizumab in cisplatin-ineligible patients with previously untreated locally advanced or metastatic urothelial cancer. J Clin Oncol.

[30] Park I, Kim BS, Lim HY (2020). Gemcitabine plus carboplatin versus gemcitabine plus oxaliplatin in cisplatin-unfit patients with advanced urothelial carcinoma: a randomised phase II study (COACH, KCSG GU10-16). Eur J Cancer.

[31] (2025). Sheng X, Zeng G, Zhang C, et al.; RC48-C016 Trial Investigators. Disitamab vedotin plus toripalimab in HER2-expressing advanced urothelial cancer. N Engl J Med.

