# Supplementary material for: A systematic review and network meta analysis of first-line immunotherapy for advanced urothelial carcinoma
Source: Cancer Drug Resist. 2026 Jun 5;9:21. doi: 10.20517/cdr.2026.22 (PMC13352428; doi:10.20517/cdr.2026.22)
Supplement: Supplementary file 1 [file cdr-9-21-SupplementaryMaterials.pdf]

Supplementary Materials

A systematic review and network meta analysis of first-line immunotherapy for advanced urothelial carcinoma

Yang Liu[#], Yuxuan Song[#], Jilin Wu, Jincong Li, Wenbo Yang, Yiqing Du, Caipeng Qin, Tao Xu

Department of Urology, Peking University People’s Hospital, Beijing 100044, China.
[#]Authors contributed equally to this work.

Correspondence to: Prof. Tao Xu, Prof. Yuxuan Song, Department of Urology, Peking University People’s Hospital, Beijing 100044, China. E-mail: xutao@pkuph.edu.cn; yuxuan_song@bjmu.edu.cn

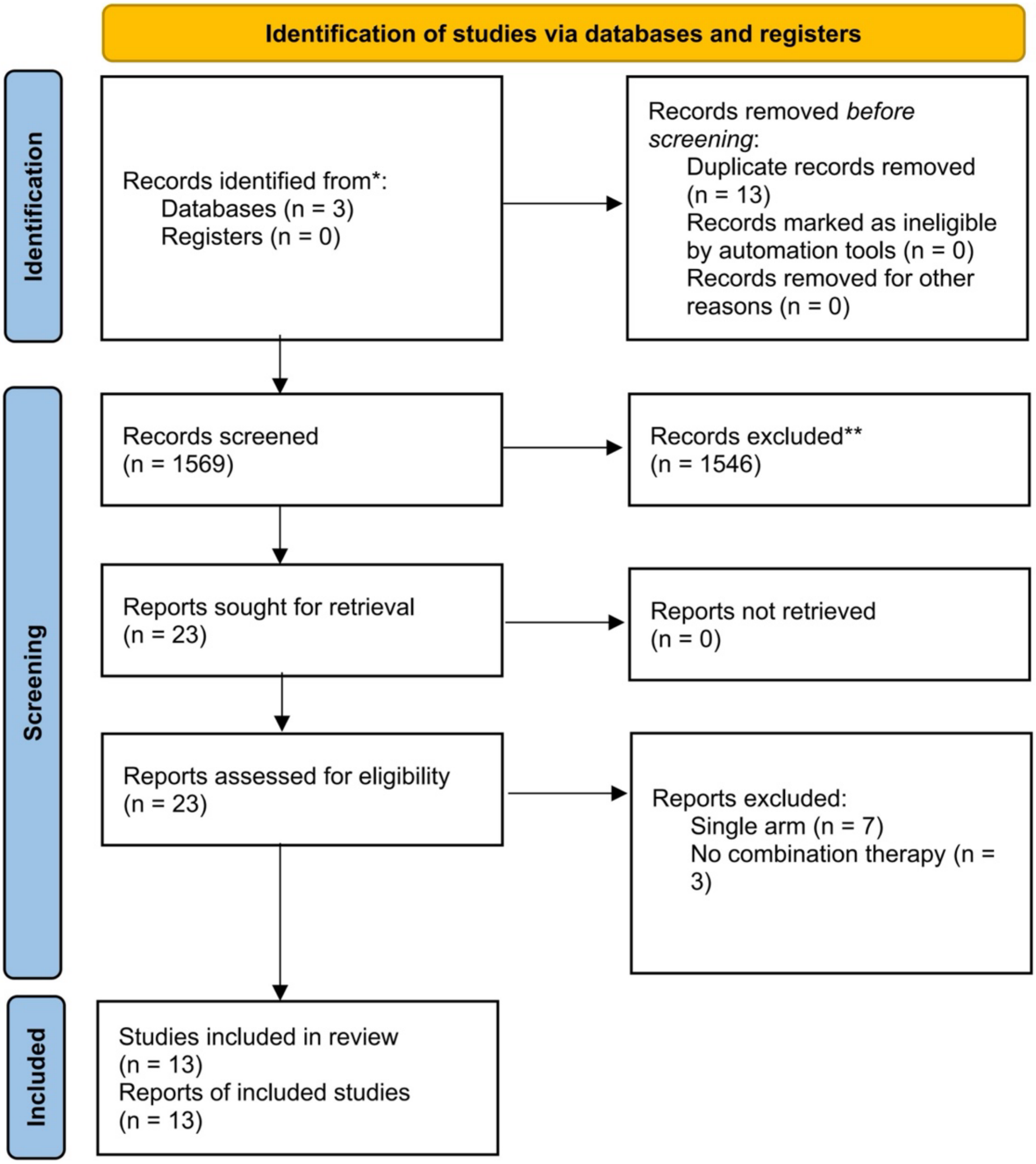

Supplementary Figure 1. PRISMA 2020 flow diagram of study selection.

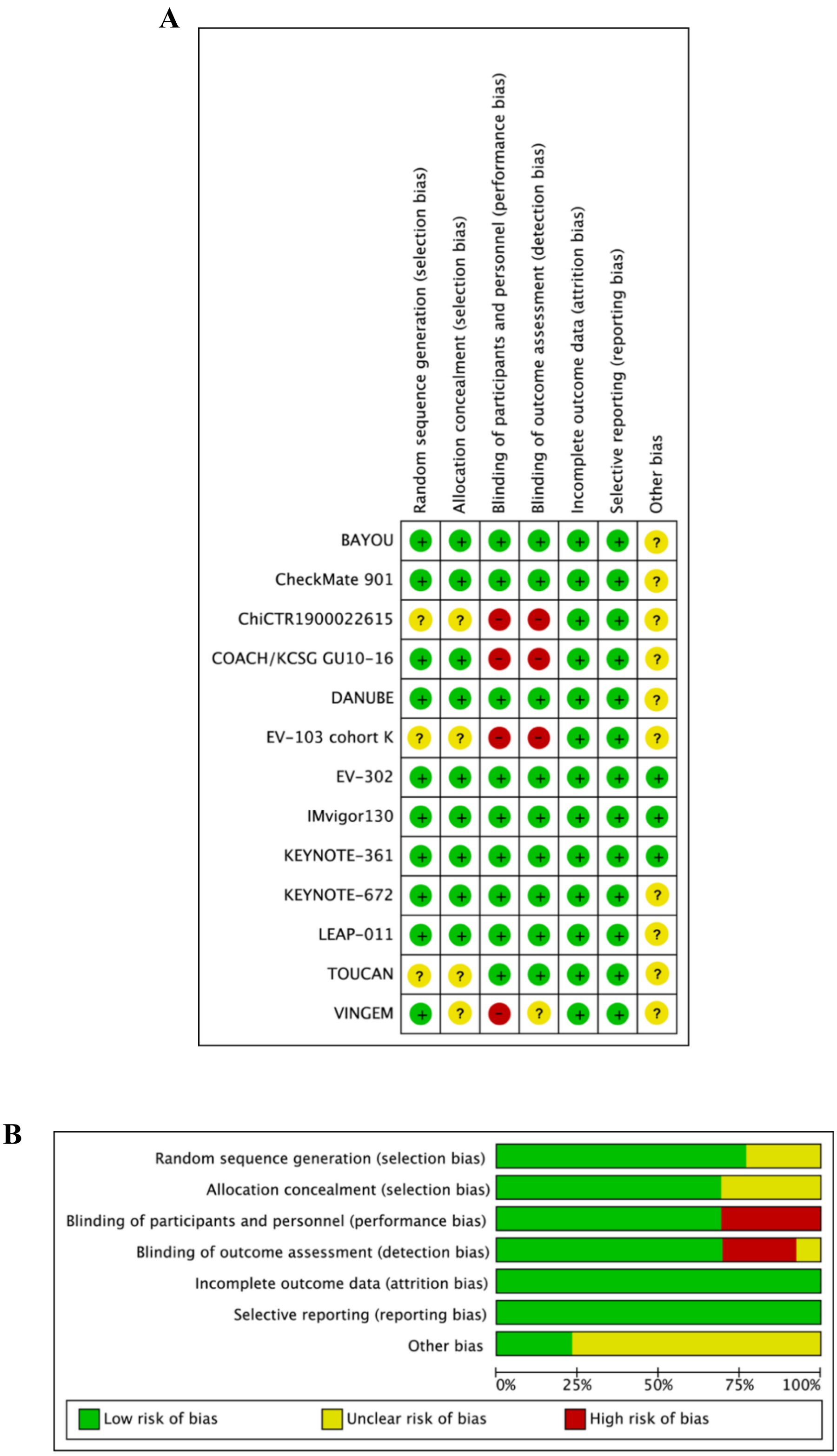

Supplementary Figure 2. (A) Risk of bias assessment for included RCTs using the RoB tool; (B) Summary of risk of bias assessment for included RCTs using the RoB tool.

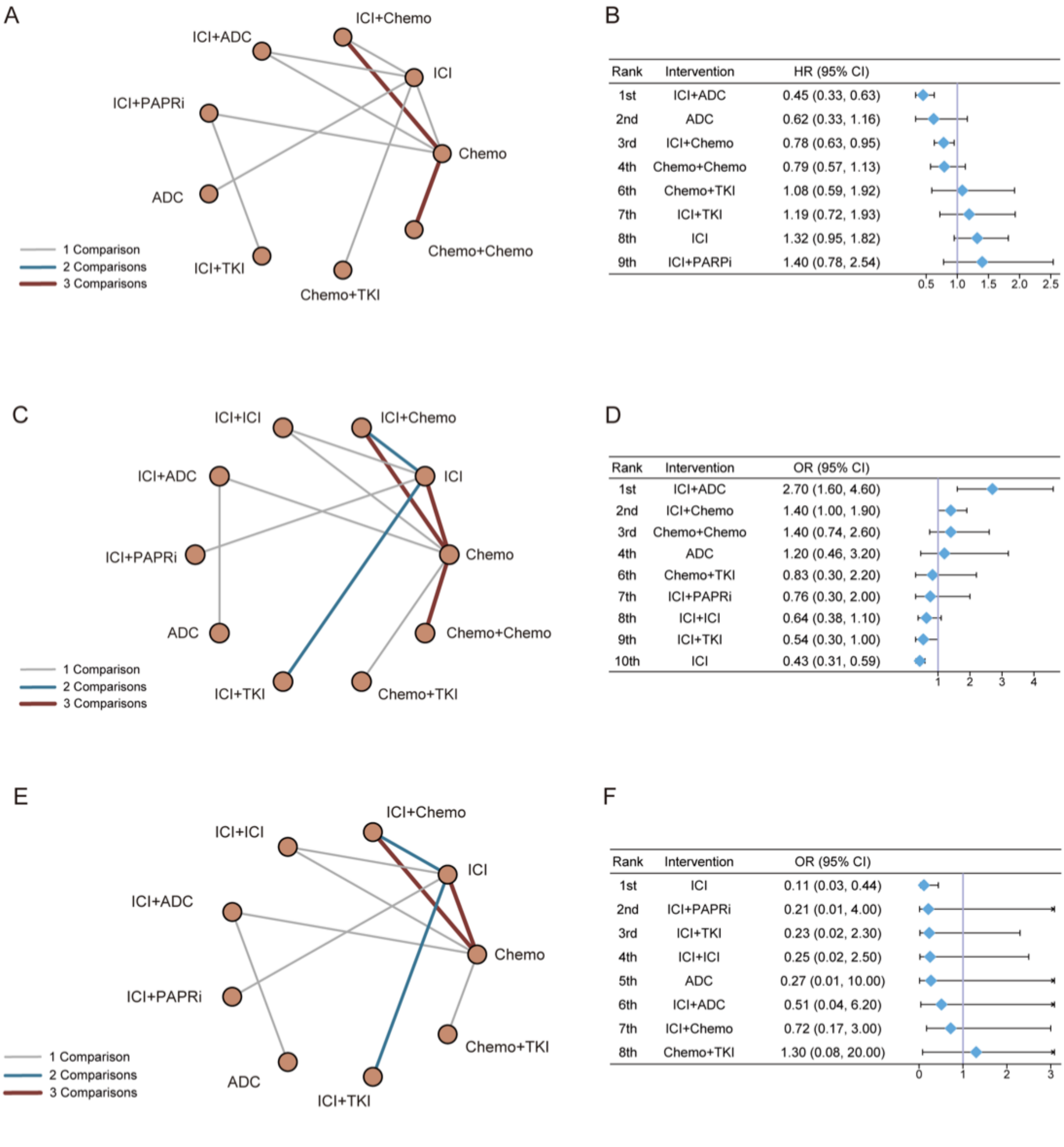

Supplementary Figure 3. Networks and forest plots for PFS, ORR, and AEs in all patients. (A) Network geometry for PFS; (B) Forest plot of PFS HRs versus chemotherapy (HR <1 favors the experimental regimen); (C) Network geometry for ORR; (D) Forest plot of ORR ORs versus chemotherapy (OR >1 indicates higher response); (E) Network geometry for grade ≥3 AEs; (F) Forest plot of AE ORs versus chemotherapy (OR <1 indicates fewer severe AEs).

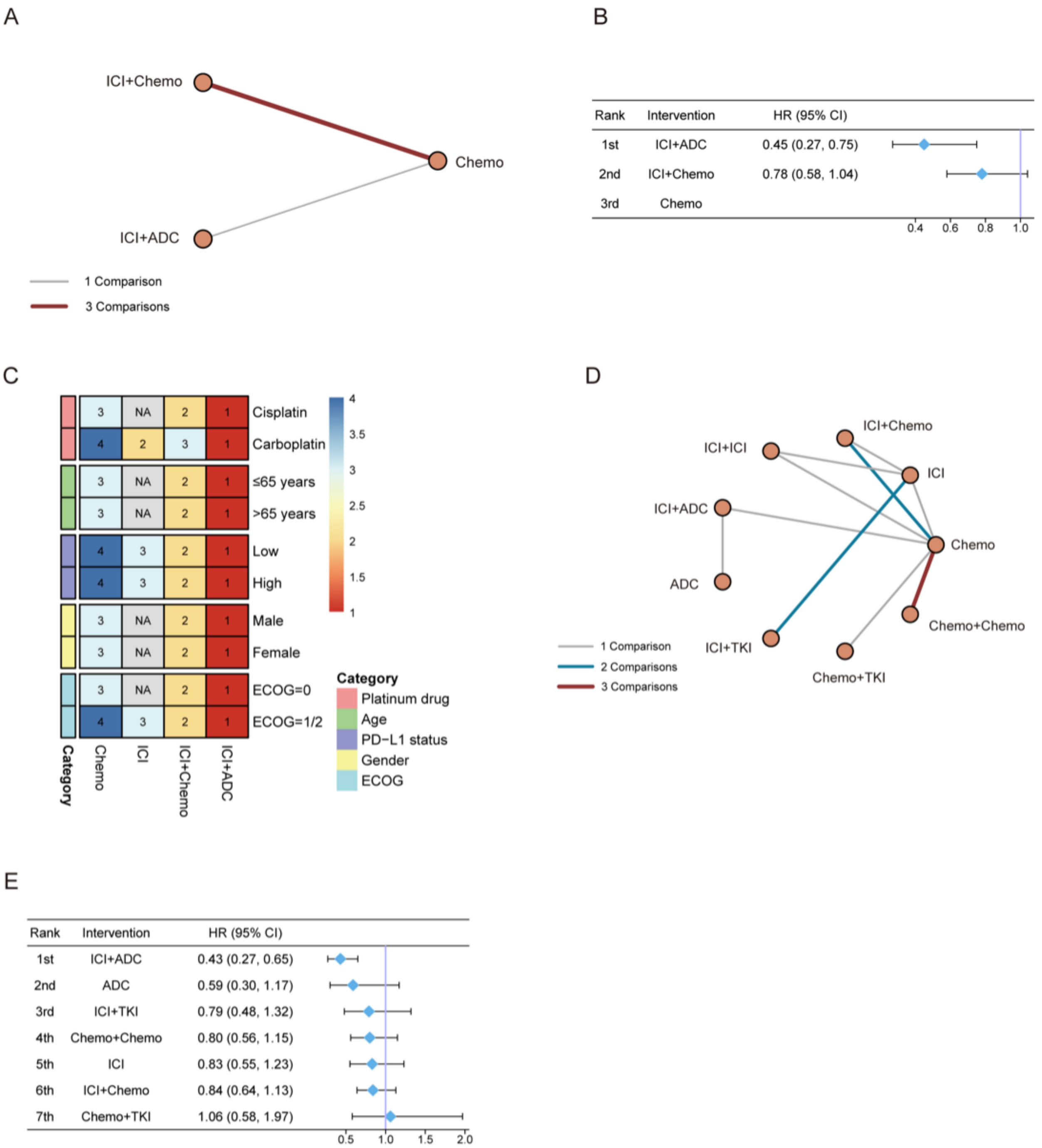

Supplementary Figure 4. PFS analyses by cisplatin eligibility and clinical subgroups. (A) Network geometry for PFS in cisplatin-eligible patients; (B) Forest plot of PFS HRs versus chemotherapy in cisplatin-eligible patients; (C) Heatmap of SUCRA-based subgroup rankings for PFS in cisplatin-eligible patients (platinum drug, age, PD-L1 status, gender, ECOG); (D) Network geometry for PFS in cisplatin-ineligible patients; (E) Forest plot of PFS HRs versus chemotherapy in cisplatin-ineligible patients.

Supplementary Table 1. Full search strategies for PubMed and Embase, with retrieval counts

Database	Search	Hits
PubMed	(urothelial carcinoma OR bladder cancer OR transitional cell carcinoma OR upper tract urothelial carcinoma OR UTUC) AND (chemotherapy OR cisplatin OR carboplatin OR gemcitabine OR docetaxel OR paclitaxel OR nab-paclitaxel OR methotrexate OR vinblastine OR doxorubicin OR MVAC OR GC OR PGC OR chemoimmunotherapy OR chemo-immunotherapy OR immunotherapy plus chemotherapy OR immunotherapy OR immune checkpoint inhibitor OR ICI OR ICIs OR ICB OR PD-1 OR PD1 OR PD-L1 OR PDL1 OR CTLA-4 OR CTLA4 OR anti-PD-1 OR anti-PD-L1 OR anti-CTLA-4 OR pembrolizumab OR nivolumab OR atezolizumab OR durvalumab OR avelumab OR tremelimumab OR ipilimumab OR keytruda OR opdivo OR tecentriq OR imfinzi OR bavencio OR yervoy OR antibody-drug conjugate OR ADC OR ADCs OR enfortumab vedotin OR EV OR padcev OR NECTIN4 OR NECTIN-4 OR sacituzumab govitecan OR SG OR trodelvy OR TROP2 OR TROP-2 OR disitamab vedotin OR RC48 OR HER2 OR ERBB2 OR trastuzumab deruxtecan OR T-DXd OR datopotamab deruxtecan OR Dato-DXd) AND (randomized controlled trial OR RCT OR phase II OR phase III) AND("first-line" OR "initial treatment" OR "untreated")	403
Embase	('urothelial carcinoma'/exp OR 'bladder cancer'/exp OR 'transitional cell carcinoma'/exp OR 'upper urinary tract cancer'/exp OR ((urothelial NEXT/1 carcinoma) OR (transitional NEXT/1 cell NEXT/1 carcinoma) OR (upper	1047

NEXT/1 tract NEXT/1 urothelial NEXT/1 carcinoma) OR UTUC OR (bladder NEXT/1 cancer) OR (bladder NEXT/1 carcinoma)):ti,ab,kw) AND (chemotherapy:ti,ab,kw OR chemotherap*:ti,ab,kw OR cisplatin:ti,ab,kw OR carboplatin:ti,ab,kw OR gemcitabine:ti,ab,kw OR docetaxel:ti,ab,kw OR paclitaxel:ti,ab,kw OR (nab NEXT/1 paclitaxel):ti,ab,kw OR methotrexate:ti,ab,kw OR vinblastine:ti,ab,kw OR doxorubicin:ti,ab,kw OR MVAC:ti,ab,kw OR (gemcitabine NEAR/3 cisplatin):ti,ab,kw OR PGC:ti,ab,kw OR chemoimmunotherapy:ti,ab,kw OR 'chemo-immunotherapy':ti,ab,kw OR (immunotherapy NEAR/3 chemotherapy):ti,ab,kw OR immunotherapy:ti,ab,kw OR ('immune checkpoint inhibitor*':ti,ab,kw OR ICI:ti,ab,kw OR ICIs:ti,ab,kw OR ICB:ti,ab,kw) OR ('PD-1':ti,ab,kw OR PD1:ti,ab,kw OR 'PD-L1':ti,ab,kw OR PDL1:ti,ab,kw OR 'CTLA-4':ti,ab,kw OR CTLA4:ti,ab,kw OR pembrolizumab:ti,ab,kw OR nivolumab:ti,ab,kw OR atezolizumab:ti,ab,kw OR durvalumab:ti,ab,kw OR avelumab:ti,ab,kw OR tremelimumab:ti,ab,kw OR ipilimumab:ti,ab,kw OR keytruda:ti,ab,kw OR opdivo:ti,ab,kw OR tecentriq:ti,ab,kw OR imfinzi:ti,ab,kw OR bavencio:ti,ab,kw OR yervoy:ti,ab,kw) OR ('antibody drug conjugate':ti,ab,kw OR 'antibody-drug conjugate':ti,ab,kw OR ADC:ti,ab,kw OR ADCs:ti,ab,kw OR 'enfortumab vedotin':ti,ab,kw OR EV:ti,ab,kw OR padcev:ti,ab,kw OR NECTIN4:ti,ab,kw OR 'NECTIN-4':ti,ab,kw OR 'sacituzumab govitecan':ti,ab,kw OR SG:ti,ab,kw OR trodelvy:ti,ab,kw OR TROP2:ti,ab,kw OR 'TROP-2':ti,ab,kw OR 'disitamab vedotin':ti,ab,kw OR RC48:ti,ab,kw OR HER2:ti,ab,kw OR ERBB2:ti,ab,kw OR 'trastuzumab deruxtecan':ti,ab,kw OR 'T-DXd':ti,ab,kw OR 'datopotamab deruxtecan':ti,ab,kw OR 'Dato-DXd':ti,ab,kw)) AND

	('randomized controlled trial'/exp OR 'phase 2 clinical trial'/exp OR 'phase 3 clinical trial'/exp OR random*:ti,ab,kw OR (double NEAR/1 blind*):ti,ab,kw OR (single NEAR/1 blind*):ti,ab,kw) AND ('first-line':ti,ab,kw OR (first NEXT/1 line):ti,ab,kw OR 'initial treatment':ti,ab,kw OR untreated:ti,ab,kw OR 'treatment-naive':ti,ab,kw OR 'treatment naïve':ti,ab,kw) AND [english]/lim AND [humans]/lim AND [1974-2025]/py	
Cochrane Library	#1 MeSH descriptor: [Urinary Bladder Neoplasms] explode all trees<br>#2 MeSH descriptor: [Carcinoma, Transitional Cell] explode all trees<br>#3 MeSH descriptor: [Ureteral Neoplasms] explode all trees<br>#4 (urothelial NEAR/3 carcinoma):ti,ab,kw<br>OR (transitional NEAR/2 cell NEAR/2 carcinoma):ti,ab,kw<br>OR (upper NEAR/2 tract NEAR/2 urothelial NEAR/2 carcinoma):ti,ab,kw<br>OR UTUC:ti,ab,kw<br>OR (bladder NEAR/1 cancer):ti,ab,kw<br>OR (bladder NEAR/1 carcinoma):ti,ab,kw<br>#5 #1 OR #2 OR #3 OR #4<br>#6 MeSH descriptor: [Antineoplastic Combined Chemotherapy Protocols] explode all trees<br>#7 MeSH descriptor: [Immunotherapy] explode all trees	132

#8 MeSH descriptor: [Immunoconjugates] explode all trees

#9 MeSH descriptor: [Programmed Cell Death 1 Receptor] explode all trees

#10 MeSH descriptor: [B7-H1 Antigen] explode all trees

#11 MeSH descriptor: [CTLA-4 Antigen] explode all trees

#12 MeSH descriptor: [Cisplatin] explode all trees

#13 MeSH descriptor: [Carboplatin] explode all trees

#14 MeSH descriptor: [Gemcitabine] explode all trees

#15 #6 OR #7 OR #8 OR #9 OR #10 OR #11 OR #12 OR #13 OR #14

#16 (random* OR placebo OR double NEXT/1 blind* OR single NEXT/1 blind*
OR phase NEXT/1 2 OR phase NEXT/1 II OR phase NEXT/1 3 OR phase NEXT/1 III
OR trial):ti,ab,kw

#17 ("first-line" OR (first NEAR/1 line) OR "initial treatment"
OR untreated OR "treatment-naive" OR "treatment naïve"):ti,ab,kw

#18 #5 AND #15 AND #16 AND #17

Supplementary Table 2. Comparison of current study with previous Meta-analyses

Items	Chen et al. 2021 [1]	Chierigo et al. 2022 [2]	Li et al. 2022 [3]	Mamede et al. 2024 [4]	Monteiro et al. 2024 [5]	Present study
**Final time for research**	October, 2020	March, 2021	June, 2021	January, 2024	January, 2024	November, 2025
**No. of included studies**	3 RCTs (13 reports)	5 RCTs	3 trials	3 RCTs (4 reports)	5 studies	13 RCTs
**Whether included Conference Abstract or ongoing trials (No. of Conference Abstract or ongoing trials)**	Yes (not specified)	Yes (not specified)	Yes (not specified)	No	Yes (not specified)	No
**No. of included patients**	Not reported	3255 (first-line); 1452 (second-line)	3238	2162	3734	5940

**Outcomes**	OS, PFS, ORR, grade 3–5 AEs	OS, PFS, ORR, DCR, CR, PR, SD, AEs	OS, PFS, ORR, DCR, AEs	OS, PFS, ORR, safety	OS, PFS, ORR	OS, PFS, ORR, grade ≥3 AEs
**Cisplatin-stratified subgroup analysis**	Yes	No	No	No	Yes	Yes
**Phase of included RCTs**	Phase III	Phase III	Phase III	Phase III	Phase III	Phase II (5); Phase III (8)

Reference

1. Chen HL, Chan VW, Tu YK, et al. Immune Checkpoints Inhibitors and Chemotherapy as First-Line Treatment for Metastatic Urothelial Carcinoma: A Network Meta-Analysis of Randomized Phase III Clinical Trials. Cancers (Basel). 2021;13(6). [doi: 10.3390/cancers13061484.]

2. Chierigo F, Wenzel M, Würnschimmel C, et al. Immuno-oncology therapy in metastatic bladder cancer: A systematic review and network meta-analysis. Crit Rev Oncol Hematol. 2022;169:103534. [doi: 10.1016/j.critrevonc.2021.103534.]

3. Li H, Ni M, Xue C, et al. Optimal first-line treatment for platinum-eligible metastatic urothelial carcinoma: Comparison of chemo-immunotherapy, immunotherapy, and chemotherapy- A systematic review and meta-analysis. Clin Immunol. 2022;236:108927. [doi: 10.1016/j.clim.2022.108927.]

4. Mamede I, Escalante-Romero L, Celso DSG, et al. Immunotherapy Plus Chemotherapy Versus Chemotherapy Alone as First-Line Treatment for Advanced Urothelial Cancer: An Updated Systematic Review and Meta-Analysis of Randomized Controlled Trials. Clin Genitourin Cancer. 2024;22(5):102154. [doi: 10.1016/j.clgc.2024.102154.]

5. Monteiro FSM, Soares A, Mollica V, et al. Efficacy of immune checkpoint inhibitors combinations as first-line systemic treatment in patients with advanced urothelial carcinoma: A systematic review and network meta-analysis. Crit Rev Oncol Hematol. 2024;196:104321. [doi: 10.1016/j.critrevonc.2024.104321.]
